# Isolation and characterisation of *Leishmania (Leishmania) infantum* from cutaneous leishmaniasis patients in northeast Brazil

**DOI:** 10.1590/0074-02760240026

**Published:** 2024-07-08

**Authors:** Gustavo Henrique Corrêa Soares, Gustavo Rolim Barbosa, Ana Jessica Sousa Coelho, Giovanna Bedin Caetano, Conceição de Maria Pedrozo e Silva de Azevedo, Adriano Cappellazzo Coelho, Mayara Ingrid Souza Lima, Beatriz Simonsen Stolf

**Affiliations:** 1 Universidade de São Paulo, Instituto de Ciências Biomédicas, Departamento de Parasitologia, São Paulo, SP, Brasil; 2 Universidade Federal do Maranhão, Centro de Ciências Biológicas e da Saúde, Departamento de Biologia, São Luís, MA, Brasil; 3 Universidade Estadual de Campinas, Instituto de Biologia, Departamento de Biologia Animal, Campinas, SP, Brasil; 4 Universidade Federal do Maranhão, Centro de Ciências Biológicas e da Saúde, Departamento de Medicina, São Luís, MA, Brasil

**Keywords:** atypical skin lesions, clinical isolates, infectivity, tegumentary leishmaniasis

## Abstract

**BACKGROUND:**

In Brazil, *Leishmania (Leishmania) infantum* is a widely distributed protozoan parasite. The human leishmaniasis caused by this species is often associated with visceral form. Tegumentary leishmaniasis (TL) cases due to *L. (L.) infantum* in the country are considered rare but may be underestimated. Although probably uncommon, these cases represent a new challenge to the prevention and control of leishmaniasis.

**OBJECTIVES:**

Here, we describe two distinct cases of TL with atypical clinical presentations caused by *L. (L.) infantum*.

**METHODS AND FINDINGS:**

Parasites were isolated from cutaneous lesions of the two patients and typed as *L. (L.) infantum* after sequencing of the ribosomal DNA internal transcribed spacer. The dermotropic *L. (L.) infantum* isolates were compared in terms of growth culture patterns, metacyclogenesis and *in vitro* infectivity in macrophages.

**MAIN CONCLUSIONS:**

This study addresses the emergence of *L. (L.) infantum* as a causative agent of cutaneous disease in a visceral leishmaniasis hotspot located in northeast Brazil. The data presented provides novel information about the presence of dermotropic *L. (L.) infantum* in the country and demonstrates the infectivity potential of theses isolates.

Leishmaniasis is a vector-borne disease caused by *Leishmania* protozoan parasites. More than 30 *Leishmania* species infect mammals,^1^ and around 22 of them are potentially pathogenic to humans.^2^ Species from subgenera *Leishmania* and *Viannia* are the main agents of human leishmaniasis,^3^ and cause two clinical forms: tegumentary leishmaniasis (cutaneous and mucosal) (TL) and visceral leishmaniasis (VL).^4^ TL exhibits a wide clinical pleomorphism in humans, depending on the *Leishmania* species and on the immunological status of host.^3^ In the Americas, *L. (L.) amazonensis* and *L. (V.) braziliensis* are responsible for most of TL cases, while *L. (L.) infantum* is the main etiological agent of VL.^1^ ^,^ ^5^ However, there are exceptions to these classical associations.^6^ ^,^ ^7^ ^,^ ^8^

The symptomatic infections caused by *L. (L.) infantum* are usually systemic and progressively affect liver and lymphoid organs (*e.g.*, bone marrow and spleen), leading most patients to death if left untreated.^9^ Eventually, *L. (L.) infantum* can also display a dermotropic profile, leading to ulcerated or non-ulcerated cutaneous lesions.^10^ ^,^ ^11^ ^,^ ^12^ TL due to *L. (L.) infantum* has been regarded as a mild disease. Nonetheless, the infection has potential to persist and progress into a chronic course.^7^ ^,^ ^13^

TL caused by *L. (L.) infantum* seems to be more prevalent in the Mediterranean Basin,^12^ ^,^ ^14^ ^,^ ^15^ in the Middle East^16^ ^,^ ^17^ and in Central America.^11^ ^,^ ^18^ ^,^ ^19^ The prevalence of TL cases caused by this species in South America is unknown and requires further investigation. In Brazil, it is believed that most symptomatic human infections by *L. (L.) infantum* manifest in the visceral form.^20^ Reported TL cases by *L. (L.) infantum* are rare, but the lack of species identification in the routine of TL diagnosis probably leads to a huge underestimation of the real numbers. Indeed, only a few cases of this association are documented, and most reports provide only clinical and parasitological data.^21^ ^,^ ^22^ ^,^ ^23^ ^,^ ^24^

The mechanisms responsible for the dermis persistence and cutaneous infection by *L. (L.) infantum* are not understood. The immunological background of the host could influence tissue tropism to the viscera or dermis.^25^ It was reported that individuals infected with *L. (L.) infantum* who developed cutaneous lesions exhibit a strong delayed-type hypersensitivity (DTH) skin response to *Leishmania* antigens.^7^ Parasite-specific factors can also contribute to the dynamics of *L. (L.) infantum* infection, and probably to the clinical manifestation.^26^ For instance, it was shown that lipophosphoglycan (LPG) from viscerotropic strains of *L. (L.) infantum* exhibits an immunosuppressive action on macrophages, whereas LPG from dermotropic strains triggers a pro-inflammatory activity.^27^ ^,^ ^28^ Thus, factors derived from both the host and the parasite probably contribute to the clinical manifestation of *L. (L.) infantum* infections.

Analysing *Leishmania* isolates from patients is a powerful approach for understanding the clinical outcomes of infection.^29^ ^,^ ^30^ ^,^ ^31^ ^,^ ^32^ Here, we describe the isolation, molecular typing and analysis of infectivity of two *L. (L.) infantum* clinical isolates from cutaneous leishmaniasis cases in northeast Brazil.

## SUBJECTS AND METHODS

*Ethics statement* - Skin biopsies and data were obtained from patients that participate in a broad study conducted by the Federal University of Maranhão. All procedures were performed according to approved guidelines and regulations of Comitê de Ética em Pesquisa da Universidade Federal do Maranhão (Process No. 3.921.086). Written informed consent was obtained prior to skin biopsy, as reported in the Comitê de Ética em Pesquisa do Hospital Universitário Federal do Maranhão (Process No. 004372/2008-70). The clinical isolates obtained were registered in SisGen (Sistema Nacional de Gestão do Patrimônio Genético e do Conhecimento Tradicional Associado - Brazil) under the identifier ADA06AF, as determined by the Decree No. 8.722 regulated by the Ministério do Meio Ambiente e Mudança Climática (Law No. 13.123/2015).

Four- to eight-week-old female BALB/c mice were purchased from the Animal facility of Faculty of Medicine, University of São Paulo (São Paulo, Brazil). All animals were kept at the Animal Facility of the Department of Parasitology and Microbiology, Institute of Biomedical Sciences, University of São Paulo. All experimental procedures were performed according to the guidelines of Brazilian College of Animal Experimentation and the Institutional Animal Care and Use Committee (CEUA) of the Institute of Biomedical Sciences, University of São Paulo, under the number #9829290419.

*Isolation of Leishmania from skin biopsy and parasite culture* - Tissue samples of skin biopsies were obtained for parasite isolation as previously described.^33^ Briefly, tissue cell suspensions were prepared in supplemented (see below) M199 medium (Sigma-Aldrich) using a cell strainer 100 µm (Corning) and maintained at 24ºC. The suspensions were serially diluted (1:10) in 24-well plates (Costar) and examined weekly for the presence of promastigotes.

After isolation, the parasites were expanded *in vitro* in supplemented M199 medium for four-five days and subsequently cryopreserved at P1 in M199 medium supplemented with 45% heat-inactivated foetal calf serum (hiFCS) (Gibco) and 10% dimethyl sulfoxide (DMSO). The isolates were defrosted and sub-cultured once before the experiments. Promastigotes from the clinical isolates of both patients were grown at 24ºC in M199 medium supplemented with HEPES 40 mM [pH 7.4], 0.3 g/L sodium bicarbonate, adenine 100 µM, hemin 5ppm, 10% hiFCS, 2% sterile human male urine and 20 µg/mL gentamicin (Gibco). Parasite culture was synchronised^34^ and sub-cultured in an inoculum of 5 x 10^5^ promastigotes/mL. Growth curves for the two clinical isolates were determined by counting in a haemocytometer daily for seven days and calculating parasite density. For comparative purposes, we used *L. (L.) infantum* LD strain (MHOM/BR/1972/LD) obtained from a VL patient.^35^ Parasites were used up to the eighth passage as promastigotes for all experiments.

*Molecular typing* - Genomic DNA (gDNA) was isolated using phenol/chloroform/isoamyl alcohol method.^36^ For species typing, two different approaches were used: (I) multiplex polymerase chain reaction (PCR) of the kDNA minicircle^37^ and (II) amplification, cloning in pGEM-T vector, followed by Sanger sequencing of the ribosomal DNA internal transcribed spacer (ITS).^38^ The list of primers used for amplification of multiplex PCR and ITS amplification followed sequencing are listed in Table I.

**TABLE I:** List of primers used for molecular typing of the clinical isolates

Approach	Primer	Sequence	Amplicon size	Reference
Multiplex PCR kDNAminicircle	LSP-*F* LVB-*R* LLA-*R* LLC-*R*	5’- gggtaggggcgttctg -3’ 5’- gcgcggcccactata -3’ 5’- cccccagttgtgaccg -3’ 5’- ccgatttttgaacggga -3’	----- 127 bp 100 bp 60 bp	^(50)^
ITS PCR	IR1 IR2	5’- gtcgtaggtgaacctgcagcagctggatcatt -3’ 5’- gcgggtagtcctgccaaacactcaggtctg -3’	1-1.2 kb	^(51)^
ITS sequencing	M13-*F* M13-*R* 5.8S-*F* 5.8S-*R*	5’- cgccagggttttcccagtcacgac -3’ 5’- tcacacaggaaacagctatgac -3’ 5’- gcagtaaagtgcgataagtgg -3’ 5´- ggaagccaagtcatccatc -3´	--------	^(51)^

*Metacyclogenesis* - The frequency of metacyclic promastigotes on day 2, 4 and 6 was estimated by flow cytometry as previously described.^39^ Briefly, 2 x 10^6^ promastigotes were collected, washed twice with phosphate-buffered saline (PBS) (800 xg, 10 min at 4ºC) and fixed in 0,5% paraformaldehyde in PBS for 30 min at 4ºC. Next, the cells were washed with PBS and cell acquisition was performed in FACSCalibur™ (BD Bioscience). The frequency of metacyclic promastigotes was determined with FSC^low^ gating from 25,000 events using FlowJo Software v10.8.1.

*In vitro macrophage infection* - Bone marrow-derived macrophages (BMDM) were obtained from BALB/c mice as previously described.^40^ BMDM were plated at a density of 4 x 10^5^ cells per well in RPMI 1640 medium (Gibco) supplemented with 10% hiFCS and 20 µg/mL gentamicin on coverslips in 24-well plates and incubated overnight in a 5% CO_2_ atmosphere at 37ºC. Cells were infected with early (Day 4) or late (Day 6) stationary-phase promastigotes at a ratio of 20:1 (parasites:macrophage) in a 5% CO_2_ atmosphere at 37ºC. After 4 h, non-internalised parasites were removed by washing with PBS, and RPMI 1640 medium was added. The plates were incubated under the same condition as above for 20 h. Macrophages were fixed with methanol-PBS (1:1) and stained with the Instant Prov kit (NewProv). The percentage of infected macrophages and the number of amastigotes per cell was determined by counting in one hundred macrophages per coverslip. Three independent experiments with technical triplicates were performed.

The human monocytic cell line THP-1 (ATCC TIB-202) was cultured in RPMI 1640 medium supplemented with 20% hiFCS, 2 mM L-glutamine and 20 µg/mL gentamicin in T-25 flasks. 1 x 10^5^ cells per well were differentiated in macrophage like-cell using 80 nM phorbol-12-myristate 13-acetate (PMA) for 72 h on coverslips in 24-well plates in a 5% CO_2_ atmosphere at 37ºC. THP-1 derived macrophages were infected with early stat-phase promastigotes at a ratio of 20:1 for 24 h in plates in a 5% CO_2_ atmosphere at 37ºC. The cells were fixed, stained, and analysed as described above. This assay was performed in two independent experiments with technical triplicates.

*Statistical analysis* - Data analysis was performed using the GraphPad Prism 8.0.2 software (GraphPad Software Inc, San Diego, CA). The normality of the data set was tested using the Shapiro-Wilk test. The parametric one- and two-way analysis of variance (ANOVA) tests followed by Tukey’s multiple comparison post hoc test was used to compare the laboratory strain and isolates in terms of *in vitro* infectivity and metacyclogenesis, respectively. A p-value < 0.05 was considered significant for all analyses.

## RESULTS

**Fig. 1:**
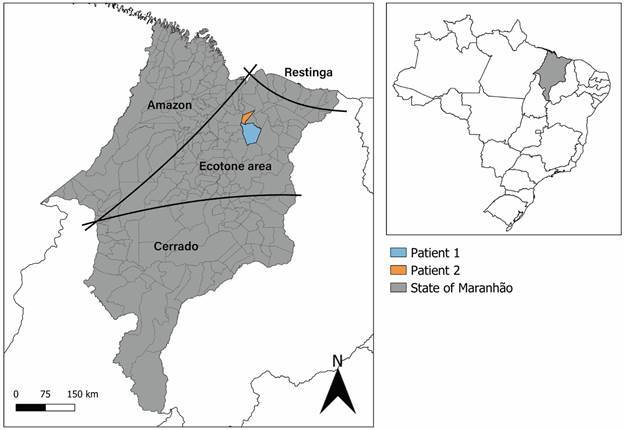
map of Brazil with the State of Maranhão highlighted. Geographical location of the patients is indicated in the map. The patients (1 and 2) reside in cities located within an ecotone region of state. Map created in QGIS v.3.24.3 (http://www.qgis.org).

*Case reports of cutaneous leishmaniasis due to Leishmania (L.) infantum* - From 2020 to 2021, two patients from an ecotone region of the State of Maranhão (Fig. 1) sought medical assistance at the reference hospital for infectious disease due to cutaneous lesions. The detailed report of each clinical case is described below.

Patient 1: A 30-year-old female patient diagnosed with human immunodeficiency virus (HIV) during her pregnancy in 2010. From 2013 to 2018, she returned for medical follow-up and received antiretroviral treatment, which included zidovudine (300 mg orally twice daily), lamivudine (150 mg orally twice daily) and efavirenz (600 mg orally once daily). However, the antiretroviral regimen was discontinued several times. In October 2020, she presented a single ulcer lesion in the lower limb [Supplementary data (Fig. 1)], and the TL diagnosis was confirmed by Giemsa-stained slides from skin scraping. She was treated daily with a dose of meglumine antimoniate (Glucantime^®^) at 20 mg/Sb^+5^/kg administered intravenously for 20 days but discontinued the treatment soon after. In late 2021, she returned to hospital service with HIV-undetectable status, but new lesions on the left lower limb and right upper limb. At that time, a biopsy sample was collected for parasite isolation, resulting in the obtaining of the clinical isolate denominated as BG03. Then, she started liposomal amphotericin B (L-AmB) therapy (3 mg/kg) for 20 days. She experienced a new relapse and started a new antimonial regimen, which was abandoned after a few days. In August 2022, the lesion recurred on the lower limb, and she started a new course of L-AmB (3 mg/kg) for 10 days, with complete healing of the lesions.

Patient 2: A 61-year-old female patient presented a single slightly painful ulcerated lesion in the right lower limb. Parasitological diagnosis on Giemsa-stained slides confirmed diagnosis of TL. She was treated daily with a dose of Glucantime^®^ at 20 mg/Sb^+5^/kg, administered intravenously for 20 days, and showed a good response, with the lesion remitting. In January 2022, the patient returned for follow-up, and no new lesions were observed. After two months, she developed ulcerated and slightly vegetative lesion on the nasal dorsum and recurrent lesions on the right lower limb. HIV serology was negative, and no other secondary infections were reported. At this point, an isolate was obtained from skin biopsy and referred to as BG05. She was treated with miltefosine (150 mg/day) for 28 days, and the lesions fully healed.

Serological screening for VL was conducted for both patients using Leishmaniose VH Bio test (Bioclin^®^), and the results were negative. Additionally, neither of the patients exhibited clinical signs of visceral disease such as hepatosplenomegaly or alterations in blood count tests. Table II summarises the main clinical characteristics of the patients.

**TABLE II:** Clinical features of tegumentary leishmaniasis patients

Patient	Field isolate code^*a*^	Lesions^*b*^		HIV-serology	Treatment^*c*^	
		Number	Type		Before isolation	After isolation
Patient 1	MHOM/BR/2021/BG03	1	Ulcerated	Positive	Glucantime^®^; L-AmB	L-AmB
Patient 2	MHOM/BR/2022/BG05	8	Ulcerated and vegetative	Negative	Glucantime^®^	MT

a: international code of *Leishmania* isolates described in this study; *b*: data collected during the skin biopsy for *Leishmania* isolation; *c*: antileishmanial drugs; Glucantime^®^: meglumine antimoniate; L-AmB: liposomal amphotericin B; MT: miltefosine.

**Fig. 2:**
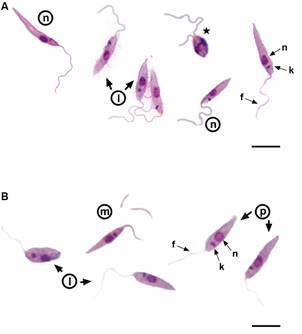
light microscopy of Giemsa-stained preparations of BG03 (A) and BG05 (B) parasites showing different promastigote morphotypes: leptomonad (l) metacylic (m) nectomonad (n), procyclic (p) and dividing-procyclics. n: nucleus; k: kinetoplast; f: flagellum. Scale-bars: 10 µm.

**Fig. 3:**
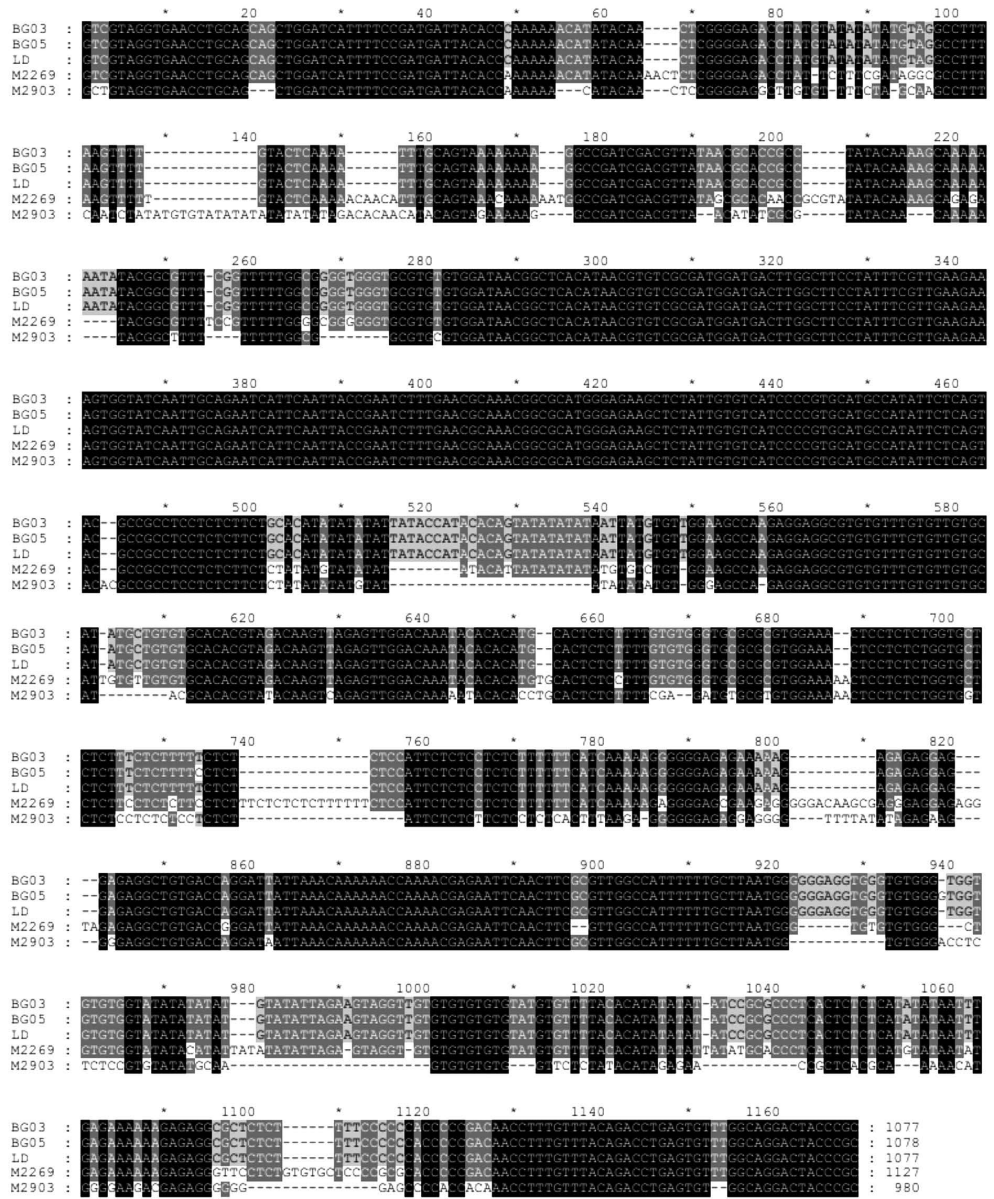
sequence alignment of the ITS of BG03 and BG05 isolates. Nucleotide sequences of *Leishmania (Leishmania) infantum* (MHOM/BR/1972/LD), *L. (L.) amazonensis* (MHOM/BR/1973/M2269) and *L. (V.) braziliensis* (MHOM/BR/1975/M2309) are included in the aligment and were obtained from GenBank (acession numbers MW538642, AJ000316.1 and AJ300483.1, respectively). The similarity between the nucleotides is highlighted in greyscale. The multiple sequence alignment was performed using GeneDoc version 2.7.

Promastigote cultures were established from each clinical isolate (Fig. 2), and the gDNA was isolated for molecular typing. We first performed a multiplex PCR targeting the kDNA minicircle as previously described^37^ and the PCR analysis indicated that both isolates belong to the *L. (L.) donovani* complex [Supplementary data (Fig. 2)]. This data was unexpected, and we thus confirmed the species of isolates by nucleotide sequencing of the ITS. The full nucleotide sequences of BG03 and BG05 showed at least 99.8% identity with *L. (L.) infantum* LD strain sequence, as well as 99.8% identity to each other (Fig. 3). Sequence data for BG03 and BG05 isolates were deposited at GenBank under accession number PP002181 and PP002182, respectively.

**Fig. 4:**
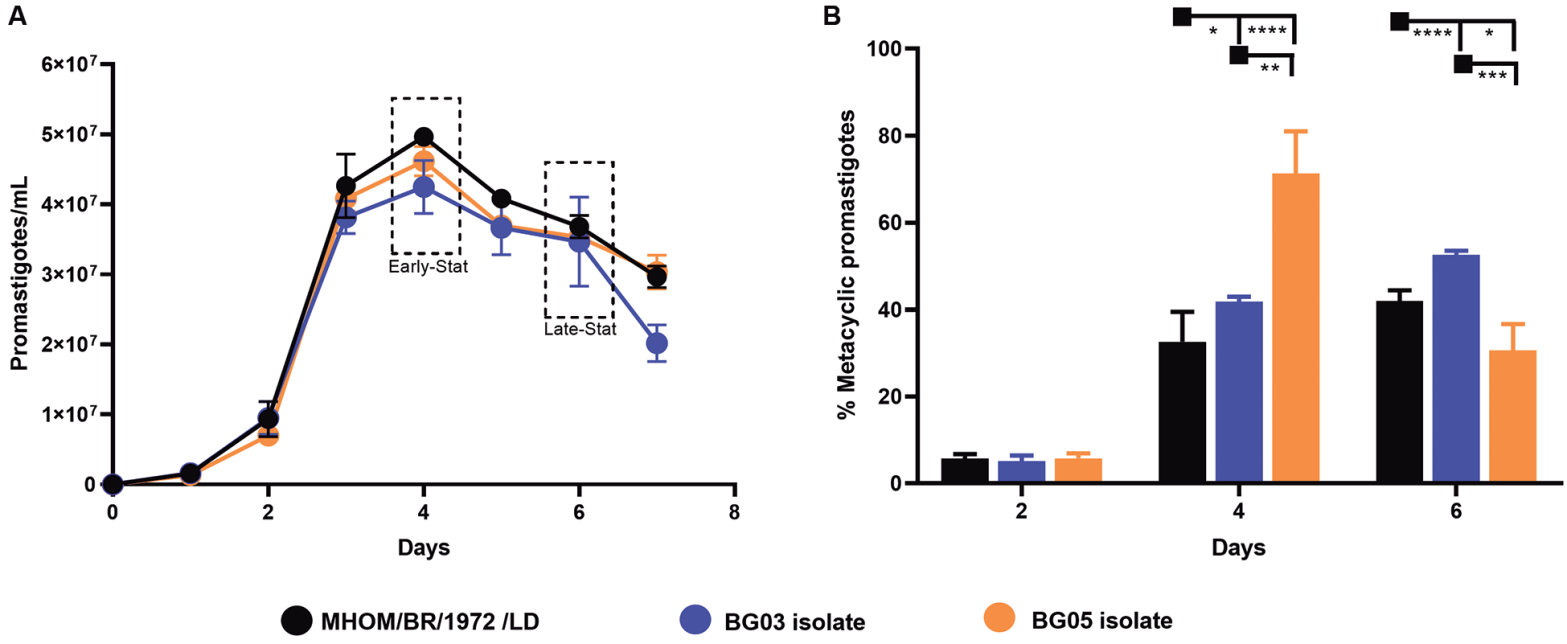
*in vitro* development of *Leishmania (Leishmania) infantum* LD strain and BG03, BG05 clinical isolates. Growth curve (A) and metacyclogenesis profile (B) of the strain and isolates. Results from three and two independent experiments with technical triplicates, respectively; data represent means ± standard error of the mean (SEM). Statistical analysis was performed by two-way analysis of variance (ANOVA).

*In vitro growth kinetics and metacyclogenesis profile of L. (L.) infantum isolates* - We then characterized the *in vitro* development of the clinical isolates. First, we determined growth curve of the parasites for seven days, starting with an initial inoculum of 5 x 10^5^ promastigotes/mL. All growth curves showed similar profiles, in which promastigotes grew logarithmically on days 2 and 3 and reached the stationary phase on day 4 (Fig. 4A). No statistically significant differences were observed in cell densities between LD strain and BG03 and BG05 isolates (Fig. 4A).

Next, we compared the estimated frequencies of metacyclic promastigotes in early log. phase (2nd day), early- and late stat. phase (4th and 6th day, respectively) cultures by flow cytometry. Size analysis of parasites revealed two different populations: non-metacyclic forms (FSC^high^) and metacyclic forms (FSC^low^) (see gating strategy on Supplementary data (Fig. 3). We used parasites on the 2nd day of culture as negative control for metacyclogenesis, given the low frequency of metacyclic forms observed during the log. phase of growth.^41^ ^,^ ^42^ No differences were found on day 2 between the LD strain and the two clinical isolates (Fig. 4B). Metacyclogenesis analysis revealed different patterns for BG03 and BG05 on days- 4 and 6. The frequency of metacyclic promastigotes for LD strain and BG03 increased slightly from fourth to sixth day of culture (Fig. 4B). Interestingly, the BG05 isolate showed higher frequency of metacyclic forms on the fourth day compared to LD strain and BG03 isolate, but this frequency drastically decreased on the sixth day (Fig. 4B).

**Fig. 5:**
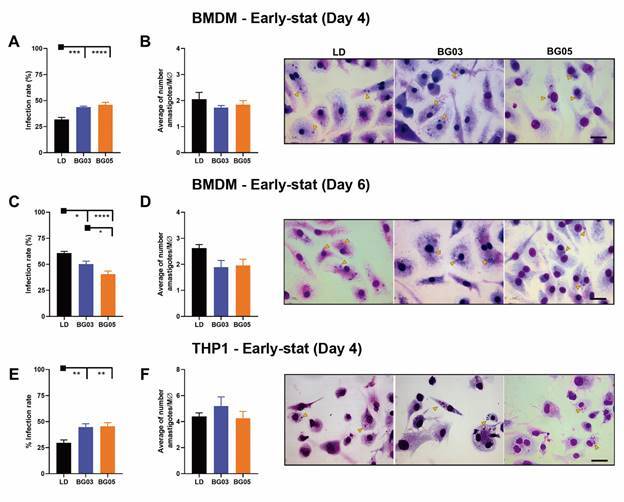
*in vitro* infectivitiy of *Leishmania (Leishmania) infantum* BG03 and BG05 isolates and MHOM/BR/1972/LD strain in distict macrophages. *In vitro* infection of bone marrow-derived macrophages (BMDM) with early-stationary (A, B) and late-stationary (C, D) promastigotes. Results from three independent experiments for BMDM in triplicate and data represent means ± standard error of the mean (SEM). *In vitro* infection of human monocyte-derived macrophage with early-stationary promastigotes (E, F). Results from two independent experiments for macrophage-like THP-1 and data represent means ± SEM. Photomicrograph of BMDM and macrophage-like THP-1 cells infected with *L. infantum* laboratory strain and isolates on day- 4 and 6 of culture. Yellow arrow indicates intracellular amastigotes. Scale-bars: 10 µm. Statistical analysis was performed by one-way analysis of variance (ANOVA) followed by Tukey’s multiple comparison post-hoc test.

*In vitro infectivity of L. (L.) infantum isolates* - To compare the infectivity of *L. (L.) infantum* isolates with the reference strain, we infected BMDM with early- and late stationary-phase promastigotes at 20:1 parasite-to-cell ratio. The percentage of infected macrophages with early stat-phase promastigotes was 43.7 ± 1.0% and 45.8 ± 2.4% for BG03 and BG05 isolates, respectively. This data showed that promastigotes of the clinical isolates are more infective on day 4 compared to *L. (L.) infantum* LD strain (31.9 ± 1.9%) (Fig. 5A). No statistically significant differences were observed in the average of number of amastigotes per macrophage between the LD strain and the clinical isolates using early stat-phase promastigotes (Fig. 5B).

Different results were observed for infections with late stat-phase promastigotes (Fig. 5C). LD strain was the most infective (60.7 ± 1.7%), while the percentage of infected macrophages for BG03 and BG05 isolates was 50.1 ± 2.9% and 40.5 ± 2.9%, respectively (Fig. 5C). An increase in the average number of amastigotes per cell was observed when using promastigotes on day 6 compared to day 4 (Fig. 5B-D). However, no statistically significant differences were observed in the number of amastigotes per macrophage between LD strain and clinical isolates using late stat-phase promastigotes (Fig. 5D).

We also investigated the *in vitro* infectivity of LD strain and clinical isolates in macrophage-like THP-1 cells using early stat-phase promastigotes. A similar infection profile in THP-1-derived macrophages was observed using early-stat promastigotes, where BG03 (44.8 ± 3.1%) and BG05 (45.5 ± 3.4%) were more infective compared to LD strain (29.7 ± 2.8%) (Fig. 5E). Although there was no significant difference in the number of amastigotes per macrophage observed in this cell model, the average parasite count was slightly higher compared to BMDM (Fig. 5B, F).

## DISCUSSION

In Brazil, infections caused by *L. (L.) infantum* often lead to visceral leishmaniasis,^20^ a clinical manifestation that begins with the spread of the parasite from the site of inoculation to the lymphoid tissues by lymphohematogenous pathway.^43^ Although less common, *L. (L.) infantum* can remain in the dermis and cause a skin infection. The mechanisms behind this phenomenon are poorly understood, but it is known that *L. (L.) donovani*, another viscerotropic species, is also capable of causing skin disease.^44^ In Central America, especially in Honduras, *L. (L.) infantum* is responsible for a cutaneous manifestation denominated non-ulcerated cutaneous leishmaniasis (NUCL) or atypical dermal leishmaniasis (ADL).^11^

Few cases of TL by *L. (L.) infantum* were reported in Brazil.^21^ ^,^ ^22^ ^,^ ^23^ ^,^ ^24^ In 1986, the first case of TL due to *L. (L.) infantum* was described in the State of Rio de Janeiro, southeast Brazil, from human and canine cases with active cutaneous lesion.^21^ Subsequently, similar cases were documented in the states of Minas Gerais^22^ and Mato Grosso do Sul.^24^ The limited description of these cases prompt us to question whether cutaneous manifestation caused by *L. (L.) infantum* are truly uncommon in the country, or if their prevalence is underestimated due to the limitation of the methods used in the clinical diagnostic, which are not species-specific.

To our best knowledge, we describe for the first time two cases of TL associated with *L. (L.) infantum* infection from northeast Brazil. The State of Maranhão is a highly endemic area for leishmaniasis. From 2020 to 2022, the Ministry of Health reported a total of 3,604 cases of TL and 803 cases of VL in the state.^45^ The state harbours a great diversity of *Leishmania* species,^46^ with *L. (L.) amazonensis* ^33^ ^,^ ^47^ ^,^ ^48^ and *L. (L.) infantum* ^49^ ^,^ ^50^ being the most prevalent species in human infections. These two cases described here are autochthonous from an area where cases of TL and VL overlap,^45^ and the patients are located within a transmission route of *L. (L.) infantum*.^49^

The patients had skin lesions of the ulcerative and vegetative type on exposed areas of the body. These lesions differ from those described for NUCL lesions, which are small and with papular or nodular appearance that do not ulcer.^13^ The ulcer development in skin lesions caused by *L. (L.) infantum* has already been described in a Paraguayan patient^51^ and in other Brazilian patients.^21^ ^,^ ^24^ One of the patients displays TL/HIV coinfection (Patient 1), so we must take into account that the cutaneous manifestation may be related with immunosuppression. Indeed, in cases of TL/HIV coinfection, clinical outcomes can be atypical, exhibiting a variety of lesion types.^33^ ^,^ ^52^ ^,^ ^53^ No comorbidities that could explain the cutaneous manifestations were described in the second patient (Patient 2), reinforcing the role of other host and/or parasite factors in the clinical outcome.

Few studies have addressed the biological features of Brazilian dermotropic *L. (L.) infantum* isolates. In this work, we characterized their growth patterns in culture, metacyclogenesis and infectivity in macrophages, comparing with the reference LD strain. The two isolates retained typical promastigote morphology in culture medium and displayed stable growth *in vitro*. We found no differences in growth curves between the isolates and LD strain. The stationary-phase of *Leishmania* growth is frequently associated with a higher proportion of metacyclic promastigotes compared to the logarithmic phase.^54^ Therefore, we used a fast and reproducible method based on flow cytometry to estimate the approximate frequencies of metacyclic forms^34^ ^,^ ^39^ and observed an increase in the frequency of metacyclic promastigotes as strain LD and isolates shifted from log- to stat-phase.

The infectivity of *L. (L.) infantum* isolates from TL cases was analysed in previous works, which described a variable infectivity profile^11^ ^,^ ^55^ Here, we investigated the infectivity of the isolates in macrophages using early and late stat-phase parasites. We also wanted to verify if the higher percentage of metacyclics observed for BG05 isolate on day 4, and the lower percentage observed on day 6, would correspond to higher and lower infections, respectively. Interestingly, both BG03 and BG05 at day 4 infected more macrophages than LD strain, while BG05 at day 6 infected less macrophages, indicating that difference in metacyclogenesis may account for part but not all differences observed in macrophage infections. The increase in infectivity of the isolates in early stat-phase have been observed in both BMDM and macrophage-like THP-1 models.

The identification of BG03 and BG05 isolates draws attention to dermotropic infections by *L. (L.) infantum* in Brazil. We provided the first example of association of this species with cutaneous clinical outcomes in the Northeast region of the country. Moreover, our study supports the use of these parasites in both *in vitro* and *in vivo* models to investigate determinants involved in the development of cutaneous disease caused by this species.
