# Supplementary material for: Isolation and characterisation of Leishmania (Leishmania) infantum from cutaneous leishmaniasis patients in northeast Brazil
Source: Mem Inst Oswaldo Cruz. 2024 Jul 8;119:e240026. doi: 10.1590/0074-02760240026 (PMC11251414; doi:10.1590/0074-02760240026)
Supplement: Supplementary file 1 [file 1678-8060-mioc-119-e240026-s.pdf]

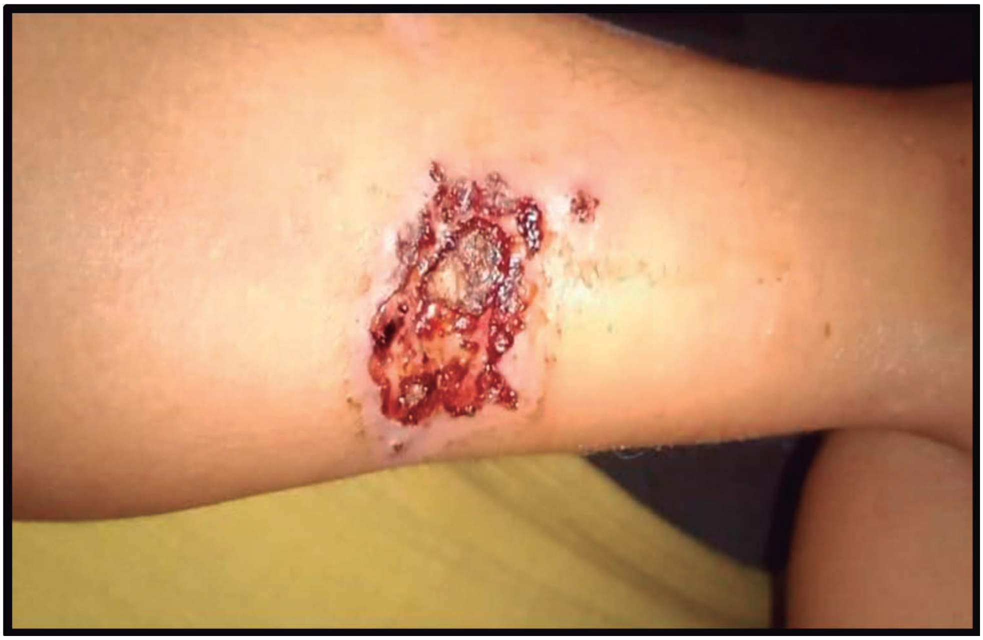

Fig. 1: atypical lesion from patient 1. Ulcerated lesion on the lower limb prior to the treatment.

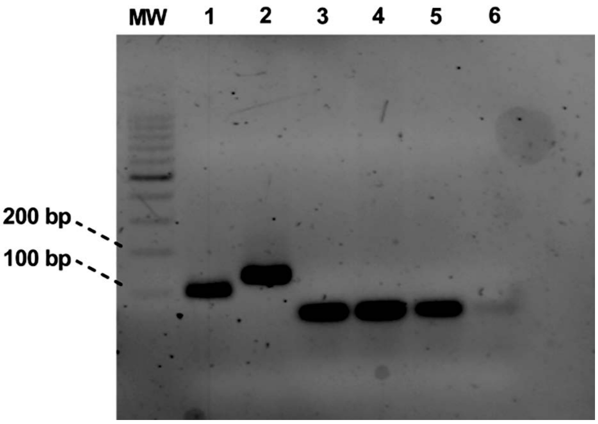

Fig. 2: genotyping of field isolates by multiplex polymerase chain reaction (PCR) of the kDNA minicircle. Analysis of PCR products by 3% agarose gel electrophoresis. MW: molecular weight 100 bp; Lane 1: *Leishmania (Leishmania) amazonensis* (MHOM/BR/1973/M2269); Lane 2: *L. (Viannia) braziliensis* (MHOM/BR/1975/M2903); Lane 3: *L. (L.) infantum* (MHOM/BR/1972/LD); Lane 4: BG03; Lane 5: BG05; Lane 6: negative control. The expected amplified fragments for *L. (L.) amazonensis*, *L. (V.) braziliensis* and *L. (L.) infantum* are 100, 127 and 60 bp, respectively.

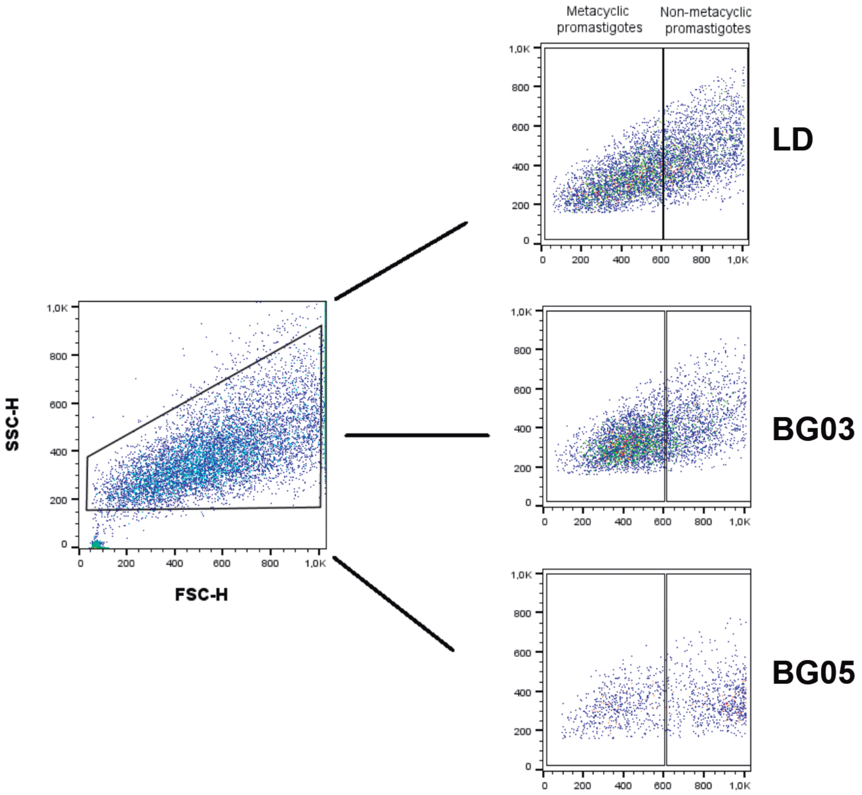

Fig. 3: metacyclogenesis evaluation by flow cytometry. Promastigotes of LD strain, BG03 and BG05 isolates were grown in M199 medium for seven days and collected on two, four and six days of culture. Representative image showing features of size (FSC) and granulocity (SSC) of parasites during late-stat phase (6th day). Gating strategy for metacyclic (FSC^{low} - left gate) and non-metacyclic (FSC^{high} - right gate) populations.
